# Sociodemographic Factors Associated with Bottle Feeding Practices in Infants Under Two Years of Age: A hospital-based study in Woldia, Ethiopia

**DOI:** 10.5195/cajgh.2020.440

**Published:** 2020-03-31

**Authors:** Yalew Mihret, Fentanesh Endalew, Hunegnaw Almaw, Melese Linger

**Affiliations:** 1 Department of Midwifery, College of Health Science, Woldia University, Woldia, Ethiopia; 2 Department of Public Health Nutrition, College of Medicine and Health Science, Bahir Dar University, Bahir Dar, Ethiopia; 3 Department of Public Health, College of Health Science, Woldia University, Woldia, Ethiopia

**Keywords:** Bottle feeding, Children, Sociodemographic factors

## Abstract

**Introduction:**

Bottle feeding should be avoided when possible in infants under the age of two to improve health outcomes. The magnitude of bottle feeding practice is currently increasing in Ethiopia, however factors associated with bottle feeding usage are rarely addressed in research. We aimed to fill this gap and assess the magnitude of bottle feeding and its association with sociodemographic factors among infants in Woldia, Ethiopia in 2019.

**Methods:**

A hospital-based cross-sectional study was conducted in Woldia General Hospital at the Immunization Clinic. A total of 255 mothers who had infants were selected by systematic random sampling method. Data was collected through face-to-face interview using a structured standardized questionnaire. The data was entered to EpiData version 3.1 and analyzed using SPSS version 20. Binary logistic regression analysis models were used to assess the association between dependent and independent variables. Variables with p-value < 0.2 in bivariable logistic regression analysis were entered to multivariable logistic regression analysis. Finally, variables with p-value < 0.05 with 95% CI in multivariable logistic regression were taken as independent predictors. COR and AOR were used to show the strength of association between the dependent and independent variables.

**Results:**

The rate of bottle feeding practice in this study was 42.7% (95%CI: 35.8, 48.2). Being an infant age 0–5 months old [AOR=0.16; 95%CI: 0.06, 0.4], being a mother age 35–50 years old [AOR=0.43; 95%CI: 0.22, 0.85], having 2–5 children [AOR=6.37; 95%CI: 1.33, 30.44], and being a farmer as reported mother's occupation [AOR=2.72; 95%CI: 1.30, 5.67] showed significant association with bottle feeding practice.

**Conclusions:**

The magnitude of bottle feeding practice was significantly higher in the current study as compared to national prevalence. Several sociodemographic factors showed significant association with bottle feeding practice which need to be explored further in the future research.

Bottle feeding is the practice of feeding an infant any substitute for breast milk with a bottle. Based on World Health Organization (WHO) classification, a prevalence of exclusive breastfeeding below 50% is considered poor.^1^ The WHO recommends that 95% of infants younger than one month and 90% of those younger than six months should be exclusively breastfed, while 90% of those aged 6–23 months should be partly breastfed. However, in low and middle-income countries, only 37% of infants younger than six months of age are exclusively breastfed.^2^ In Ethiopia, about 77% of infants scored low or medium on the breastfeeding performance index during the first six months of life,^3^ and only 58% of infants under age six months are exclusively breastfed.^4^

The period between birth up to two years of the child's life is a “critical window period” because during this period, infants are vulnerable to malnutrition or any other illness.^5^ Feeding exclusively breast milk within the first six months is considered a first vaccine for the infant.^6^ Despite breastfeeding recommendations, the prevalence and duration of breastfeeding is declining rapidly and is being replaced by bottle feeding, particularly among urban residents in developing countries.^7^ Bottle feeding is generally not recommended as it can lead to an increased incidence of excessive weight gain, diarrhea, infection, malnutrition, increased mortality, iron depletion, and decreased birth spacing.^8^^–^^15^ Even the expressed breast milk could increase infant weight gain if it is fed by the bottle.^16^

Globally, 45%, or 3.1 million, of child deaths are attributable to undernutrition annually; this problem is severe in low income countries.^14^ Increasing appropriate complementary feeding has the potential to prevent 6% of all under-5 deaths, particularly in the developing countries.^5^

Numerous studies in various countries have shown that being an urban resident,^17^,^18^ not being counseled on bottle feeding education,^18^,^19^ the infant being hospitalized,^18^ low infant age,^19^ being a homemaker, not obtaining postnatal care, lower mother's age,^19^ being an employed mother, hospital delivery, high infant age,^9^,^17^ higher maternal education level, high wealth index,^17^,^20^,^21^ as well as workload and short maternity leave^20^,^21^ were contributing factors for bottle feeding. Previous research inconsistently reported factors associated with bottle feeding practice, especially sociodemographic variables, infant age, and mothers’ educational and occupational status.

The government of Ethiopia adopted infant and young child feeding guidelines in 2004^22^ and the national nutrition program in 2013^23^ to unlock the lifesaving potential of optimal breastfeeding practices. The guideline is based on WHO recommendations that emphasizes exclusive breastfeeding. Furthermore, the health extension program in Ethiopia aims at improving proper infant and young child nutrition, including promotion of exclusive breastfeeding. ^22^

Although the government put forth the efforts mentioned above, the prevalence of bottle feeding practice among infants under two years was 11.9 %^24^ in 2011 and increased to 14% in 2016,^25^ according to Ethiopia Demographic and Health Survey Report. Therefore, this study was aimed to assess factors associated with bottle feeding practices among mothers with infants in Woldia General Hospital, Ethiopia, 2019.

## Methods

### Study setting and population

This hospital-based cross-sectional study was conducted among mothers with infants (children less than 24 months of age) who attended the immunization clinic in Woldia General Hospital, Woldia, Ethiopia. The town is located 521 kilometers away from Addis Ababa, the capital city of Ethiopia, and 360 kilometers away from Bahir Dar, the regional city of Amhara. According to the national census of 2008, the town of Woldia had a total population of 75,496, of whom 38,167 were men, and 37,279 were women. Woldia has one general hospital and two health centers. This study was conducted from February to April 2019 at Woldia General Hospital.

### Sample size and sampling technique

The sample size was calculated using a single population proportion formula: n=(Z_a/2_)2P(1-P)/w^2^ with the assumptions Z_a/2_=1.96 (95% confidence level), P=0.196 (19.6% as the prevalence of bottle feeding practice in Holeta town^19^) and w=0.05 (5% margin of error). The estimated sample size was 243. We accounted for a non-response rate of 10%, so our final sample size estimate increased to 267.

A systematic random sampling technique was conducted to select a representative population in the immunization clinic. The sampling interval (K) was established by dividing the average number of women who visited the immunization clinic in the previous three months before data collection (average n=900) by the sample size, which produced K=900/267=3. Women were recruited daily by simple random sampling technique using the lottery method, and the next respondent was selected by adding a sampling interval to the number of the selected women. The same procedure was done on subsequent days until the required sample size was reached.

### Exposure and outcome variables

The exposure variables were sociodemographic factors and obstetric factors. The definition and measurements standards of the variables were adopted from the Ethiopian Demographic and Health Survey 2016.^25^

The outcome variable was bottle feeding practice, which was categorized as yes/no. Bottle feeding practice was measured based on WHO definition of this indicator: "proportion of children 0–23 months of age that were fed any liquid (including breastmilk) or semisolid from a bottle with nipple/teat in previous 24 hours prior to data collection period".^25^

### Data collection tools and procedures

Data were collected by using a structured standard questionnaire through face-to-face interview. The questionnaire was adopted from the Ethiopian Demographic and Health Survey 2016.^25^ It has two parts: sociodemographic information and obstetric information. First, the English version of the questionnaire was prepared. It was translated to the local language of Amharic and then translated back to English. The data collectors were five graduate class midwifery students who had received one day of training before starting the data collection. The quality of the data collection process was monitored, and clear uniform instructions were given to all data collectors. All data collected as a part of this research were checked by the principal investigator.

### Data processing and analysis

Data were entered and cleaned in EpiData version 3.1, and SPSS version 20.0 was used for data analysis. Descriptive statistical analyses were employed. Binary logistic regression analysis models were used to assess the association between dependent and independent variables. Variables with p-value of < 0.2 in bivariable logistic regression analysis were entered to multivariable logistic regression analysis. Finally, variables with p-value < 0.05 with 95% CI in multivariable logistic regression were taken as independent predictors for bottle feeding practice. COR and AOR were used to show the strength of association between the dependent and independent variables

### Ethical consideration

Ethical clearance was obtained from the ethical review committee from the College of Health Science, Woldia University. The purpose of the study was explained, and confidentiality was secured by omitting any identifiers. Verbal consent was obtained from each study participant over 18 years of age. For mothers under 18 years of age, verbal consent was obtained from both, the participant and her guardian.

## Results

### Sociodemographic characteristics

From the estimated sample size, 255 participants responded to survey, (95.5% response rate). Among the study participants, 222 (87.1%) were married, 155 (60.8%) were 15–24 years of age, 138 (54.1%) were orthodox by religion, and 165 (64.7%) of mothers were urban residents (Table 1).

**Table 1.** Sociodemographic characteristics of mothers with infants less than 24 months of age in Woldia General Hospital, Ethiopia, 2019 (N=255)

Variables	Frequency	Percentage
**Age of mother**		
15–24	40	15.7%
25–34	155	60.8%
35+	60	23.5%
**Residence**		
Urban	165	64.7%
Rural	90	35.3%
**Marital status**		
Married	222	87.1%
Divorced	18	7.1%
Single	8	3.1%
Widowed	7	2.7%
**Mother's occupation**		
Homemaker	128	50.2%
Farmer	32	12.5%
Daily laborer	15	5.9 %
Merchant	39	15.3 %
Government employee	41	16.1%
**Educational status of mother**		
Unable to read and write	64	25.1 %
Primary	76	29.8%
Secondary	68	26.7%
Higher education	47	18.4%
**Religion of mother**		
Orthodox	138	54%
Muslim	91	36.2
Protestant	20	7.8%
Others	6	2%
**Occupation of husband**		
Farmer	72	28.2%
Daily laborer	21	8.2%
Merchant	86	33.7%
Government employee	76	29.8%
**Educational status of husband**		
Illiterate	38	14.9%
Primary	73	28.6%
Secondary	60	23.5%
Higher education	84	32.9%

Note: Other religions=Adventist

### Obstetric related factors

In this study, 241 (94.5%) mothers had antenatal care in their previous pregnancy. During their antenatal care follow up, 209 (87.1%) of women did not receive counseling about bottle feeding practice. From the total study participants, 218 (85.5%) received postnatal health care services for the current child (Table 2).

**Table 2.** Obstetric related factors of mothers with infants less than 24 months of age in Woldia General Hospital, Ethiopia, 2019 (N=255)

Variables	Frequency	Percent
**Gravidity of mother**		
Primigravida	70	27.5%
Multigravida	185	72.5%
**Number of children**		
1	75	29.4%
2–5	160	62.7%
6–10	20	7.8%
**Age of youngest child in months**		
0–5	55	21.6%
6–11	103	40.4%
12–23	97	38%
**Sex of infant**		
Male	163	63.9%
Female	92	36.1%
**Attended PNC for the current child**		
Yes	218	85.5%
No	37	14.5%
**Attended ANC for the last pregnancy**		
Yes	241	94.5%
No	14	5.5%
**Counseling about bottle feeding obtained**		
No	209	87.1%
Yes	31	12.9%
**Place of delivery**		
Health institution	179	70.2%
Home	76	29.8%

Note: PNC=Postnatal care, ANC=Antenatal care

### Child feeding practices

The overall rate of bottle feeding was 42.7% (95%CI: 35.8, 48.2) and the magnitude was 12.7%, 46.6% and 55.7% respectively when disaggregated into age categories of 0–5 months, 6–11 months and 12–24 months, respectively. Among the respondents who were asked about the reason why they practiced bottle feeding, around 42 (38.5%) respondents reported that they practiced bottle feeding due to work. Seventy (44.9%) anticipated practicing bottle feeding up to two years, and 176 (69.0%) previously obtained information on the benefits of breastfeeding from health professional (Table 3).

**Table 3.** Child feeding practices among mothers with infants less than 24 months of age in Woldia General Hospital, Ethiopia, 2019 (N=255)

Variables	Frequency	Percent
**Source of information on the advantage of breast feeding**		
Mass media	28	11%
Health professional	176	69%
Family	51	20%
**Type of feeding for the youngest children now**		
Only breast milk	100	39.2
Only bottle feeding	32	12.5
Breast and other foods with cup and spoon	116	45.5%
Other food with cup and spoon	7	2.7%
**Duration of bottle feeding practice**		
Up to 6 months	30	19.2%
Up to 1 year	37	23.7%
Up to 2 year	70	44.9%
Until the baby discontinues	19	12.2%
**Child bottle fed presently**		
Yes	109	42.7%
No	146	57.3%
**Reasons to start bottle feeding**		
Mothers return to work	42	38.5%
Inadequate breast milk	30	27.5%
Availability of formula milk	14	12.8%
Pregnancy	3	2.8%
Mother is ill	20	18.3%
**Time of initiation of breast feeding**		
Immediately after birth	218	85.5%
After 1 year	23	9%
When the mother feels comfortable	10	3.9%
I don't know	4	1.6%
**Breast feedings per day**		
1–7	107	42%
≥ 8	148	58%

### Health status of an infant

Among respondents who used bottles for feeding, 103 (58.9%) used formula, 18 (10.3%) of the mothers cleaned the bottle once daily, 39 (15.3%) of the infants had history of recurrent diarrhea in the past one month, and only 90 (51.4%) of the mothers cleaned the bottle through boiling (Table 4).

**Table 4.** Health status of infants less than 24 months of age in Woldia General Hospital, Ethiopia, 2019 (N=255)

Variables	Frequency	Percent
**Source of water for drinking**		
Pipe	205	80.4%
River	13	5.1%
Spring (protected)	35	13.7%
Other	2	0.8%
**History of recurrent diarrhea in the past one month**		
Yes	39	15.3%
No	216	84.7%
**History of illness in the past one month**		
Yes	20	7.9%
No	235	92.1%
**Type of illness**		
Diarrhea	5	25%
Weight loss	4	20%
Vomiting	3	15%
Abdominal pain	5	25%
Respiratory infection	3	15%
**Number of bottles**		
One	60	34.3%
Two	73	41.7%
More than two	42	24%
**Frequency of bottle cleaning**		
Every feeding pattern	91	52%
Only when spoiled	34	19.4%
Once daily	18	10.3%
Every 6 hours	32	18.3%
**Techniques of cleaning the bottle**		
Boiling	90	51.4%
Rinsing with water and soap	64	36.6%
Only rinsing with water	21	12%
**Additional food with bottle feeding**		
Yes	105	60%
No	70	40%
**Kind of fluid offering to the baby with bottle feeding**		
Cow's milk	63	36%
Formula milk	103	58.9%
Tea	2	1.1%
Expressed breast milk	7	4%

Note: Other water source=unprotected spring

### Factors associated with bottle feeding practice

In multivariable logistic regression analysis, infants aged 0–5 months old, mothers aged 35–50 years old, having 2–5 children and being a farmer as mother's occupation showed significant association with bottle feeding practice. Other factors like father's occupation, father's education, antenatal care follow up, postnatal follow up, place of delivery, mother's education level, sex of the child, parity and gravidity had no association with infant bottle feeding practices.

Infants 0–5 months old were 84% less likely to be bottle fed than infants 12–23 months old [AOR=0.16; 95%CI: 0.06, 0.4]. Furthermore, mothers who were between 35–50 years old were 57% less likely to practice bottle feeding than those who were in between ages 15–24 [AOR=0.43; 95%CI: 0.22, 0.85]. Mothers who had 2–5 children were six times as likely to practice bottle feeding as compared to mothers who have 6–10 children [AOR=6.37; 95%CI: 1.33, 30.44]. Being a farmer as mother's occupation increased the odds of bottle feeding by 2.7 times as compared to a mother who were homemakers [AOR=2.7; 95%CI: 1.30, 5.67] (Table 5).

**Table 5.** Multivariable logistic regression analyses among mothers with infants less than 24 months of age in Woldia General Hospital, Ethiopia, 2019

Variable	Bottle feeding		COR (95% CI)	AOR (95% CI)
	Yes	No		
**Mother's age in years**				
15–24	18	22	1	1
25–34	74	81	0.89 (0.446, 1.800)	0.46 (0.19, 1.10)
35–50	17	43	2.07 (0.896, 4.787)	0.43 (0.22, 0.85)
**Child's age in months**				
0–5	7	48	0.17 (0.07, 0.4)	0.16 (0.06, 0.4)
6–11	48	55	0.89 (0.51, 1.55)	0.96 (0.51, 1.8)
12–24	54	43	1	1
**Number of children**				
1	30	42	7.07 (1.53, 32.66)	4.12 (0.8, 21.19)
2–5	70	86	7.7 (1.73, 34.48)	6.37 (1.33, 30.44)
6–10	9	18	1	1
**Mother's occupation**				
Homemaker	48	80	1	1
Farmer	12	20	11 (0.45, 2.23)	2.72 (1.3, 5.67)
Daily laborer	6	9	0.9 (0.3, 2.69)	2.29 (0.87, 6.04)
Merchant	18	21	0.7 (0.34, 1.44)	2.75 (0.79, 9.24)
Government employee	25	16	0.38 (0.19, 0.79)	1.79 (0.72, 4.42)
**Mother's educational status**				
Unable to read and write	20	44	0.36 (0.19, 0.69)	4.12 (0.8, 21.19)
Primary	25	51	0.39 (0.21, 0.71)	0.27 (0.64, 1.01)
Secondary and above	64	51	1	1

Note: P-value significant at level of P<0.05, Backward LR method, Hosmer Lemeshow P-value=0.98

## Discussion

The aim of this study was to assess the magnitude of bottle feeding practice and associated factors among infants less than two years old in Woldia General Hospital. In the current study, the prevalence of bottle feeding practice was 42.7% (95%CI: 35.8, 48.2). Age of the child, age of the mother, mother's occupation, and number of children in the family were found to be associated with bottle feeding practice.

The magnitude of bottle feeding in the current study was consistent with the studies conducted in Sudan and Namibia.^17^,^18^ The magnitude of bottle feeding in this study was higher compared to the national prevalence reported in the 2016 EDHS,^25^ Holeta town,^19^ Bahir Dar^26^ and Nigeria.^27^ The possible reason for this difference might be attributed to study period variation, as there has been increasing advocacy for using formula in this region recently.

The practice of bottle feeding from the current findings was lower than in the study conducted in Ethiopia.^21^ There might also be differences due to variations in socio-cultural aspects among study participants regarding feeding practices.

Mothers in the age group of 35–50 were less likely to practice bottle feeding as compared to mothers in the age range of 15–24 years old. This finding is also supported by another study conducted in Southern Nation Nationality of People Regional State, Ethiopia, 2016.^28^ The possible explanation could that as mothers age increases, her experiences in childcare increase as well, and more appropriate care and feeding patterns are adopted.

Infants in the age group of 0–5 months old were less likely to be bottle fed than infants 12–23 months old; this finding has also been reported elsewhere^19^ and inconsistent in studies conducted in Namibia^17^ and Kenya.^9^ Infants, especially those less than 6 months of age, have gastrointestinal systems that are not well matured. Bottle feeding at this age may be associated with problems in digestion and absorption, which in turn may lead to diarrhea, vomiting, and infections. If mothers had experienced this previously, they may not start bottle feeding their child within the first year.

Being a farmer as the mother's occupation increased the likelihood of bottle feeding as compared to mothers who were homemakers. This result is consistent with a study conducted in Brazil, in which women who worked outside of home practiced bottle feeding more often.^29^ This pattern may be attributed to challenging workload among mothers who are working in farming.

This study had some limitations. The cross-sectional nature of the study may be subject to recall bias, and social desirability bias might influence the determinants of bottle feeding. For future researchers, conducting the studies with large sample size and incorporating factors such as media exposure and social media utilization will help to identify other important predictors of bottle feeding practices. The study's strength is that it used primary data and included infants up to the age of two, which helps to estimate the magnitude of bottle feeding appropriately.

Designing special nutrition intervention program for mothers who are working in farming, as well as developing a strategy to increase mothers’ education in the area of nutrition and infant health, could contribute to lower bottle feeding use and improved infant outcomes.
